# Iron Rims as an Imaging Biomarker in MS: A Systematic Mapping Review

**DOI:** 10.3390/diagnostics10110968

**Published:** 2020-11-18

**Authors:** Amjad I. AlTokhis, Abdulmajeed M. AlOtaibi, Ghadah A. Felmban, Cris S. Constantinescu, Nikos Evangelou

**Affiliations:** 1 School of Medicine, University of Nottingham, Nottingham, UK/Division of Clinical Neuroscience, Nottingham University Hospitals NHS Trust, Nottingham NG7 2UH, UK; Abdulmajeed.AlOtaibi@nottingham.ac.uk (A.M.A.); Ghadah.Felmban@nottingham.ac.uk (G.A.F.); Cris.Constantinescu@nottingham.ac.uk (C.S.C.); Nikos.Evangelou@nottingham.ac.uk (N.E.); 2 School of Health and Rehabilitation Sciences, Princess Nourah bint Abdulrahman University, Riyadh 11564, Saudi Arabia; 3 School of Applied Medical Sciences, King Saud bin Abdulaziz University, Riyadh 14611, Saudi Arabia

**Keywords:** Magnetic Resonance Imaging, Multiple Sclerosis, rims, iron rim, lesion, biomarker, imaging

## Abstract

Background: Multiple sclerosis (MS) is an autoimmune, inflammatory, demyelinating and degenerative disease of the central nervous system (CNS). To date, there is no definitive imaging biomarker for diagnosing MS. The current diagnostic criteria are mainly based on clinical relapses supported by the presence of white matter lesions (WMLs) on MRI. However, misdiagnosis of MS is still a significant clinical problem. The paramagnetic, iron rims (IRs) around white matter lesions have been proposed to be an imaging biomarker in MS. This study aimed to carry out a systematic mapping review to explore the detection of iron rim lesions (IRLs), on clinical MR scans, and describe the characteristics of IRLs presence in MS versus other MS-mimic disorders. Methods: Publications from 2001 on IRs lesions were reviewed in three databases: PubMed, Web of Science and Embase. From the initial result set 718 publications, a final total of 38 papers were selected. Results: The study revealed an increasing interest in iron/paramagnetic rims lesions studies. IRs were more frequently found in periventricular regions and appear to be absent in MS-mimics. Conclusions IR is proposed as a promising imaging biomarker for MS.

## 1. Introduction

Multiple sclerosis (MS) is an autoimmune, inflammatory, demyelinating and degenerative disease of the central nervous system (CNS) [1]. It is associated with focal inflammatory demyelinating lesions in both white and grey matter [2].

Magnetic resonance imaging (MRI) has an important role in determining the diagnosis of MS. MS diagnostic criteria using MRI are dependent on the number of spread WM lesions throughout the CNS, in both time and space [3,4]. MRI is proved to be sensitive in detecting the focal WM lesions; however, it lacks the specificity as neuroinflammation, and cerebrovascular abnormalities may mimic MS WM lesions [5,6].

To date, there is no established and uniformly used imaging biomarker for diagnosing MS, and the diagnostic criteria are based mainly on clinical relapses and the presence of lesions on MRI and oligoclonal bands in the cerebrospinal fluid. However, misdiagnosis with MS-mimicking disorders are not uncommon [7]. The presence of central vein in white matter lesions and paramagnetic iron rims (IRs) have been proposed as MRI biomarkers that can discriminate MS from non-MS disorders. These new MRI signs could potentially provide a more accurate diagnosis of MS [8,9]. This review summarizes the literature surrounding IRs in MS.

Iron rims appear as dark, ringlike features on the edges of some MS lesions have gained recent research attention [10]. Lesions with IRs are believed to be chronic active lesions, also known as smoldering or slowly expanding lesions (SELs), which fail to remyelinate and have subsequently been proposed to be a marker of tissue destruction, continuing inflammation and failure to repair [11,12,13]. Indeed, evidence from pathology and 7 Tesla (T) MRI has shown that rimmed MS lesions were found to be significantly larger and underwent expansion by almost 30%, unlike nonrimmed lesions which their size shrinks by 10% [14]. Contrarily, the nonrimmed lesions may disappear or return to a similar contrast of the normal-appearing white matter (NAWM) over time, which may affect their clinical usefulness [9,15]. 

Several studies found that IRs are commonly seen in Relapsing Remitting MS (RRMS) [16] while others found it in progressive MS. Also, some publications found IRs were more common in elderly patients with higher expanded disability status scale (EDSS score ≥5) [14,17,18], contrary to Dal-Bianco et al. who found them more common in young MS patients. Importantly, several studies reported the absence of IRs in MS mimics, such as neuromyelitis optica spectrum disorders (NMOSD) [19] Susac’s syndrome [20] and ischemic lesions [21]. This suggests that IRs might be specific features of MS lesions, which are proposed to be used not only as a diagnostic biomarker of disease progression but also diagnostically. However, initial findings from MRI studies need further validation before they can be incorporated into clinical practice. Iron rim lesions (IRLs) were significantly more often located in the periventricular regions, whereas no-IRLs were more in the juxtacorticle and Deep Gray Matter (DGM) areas.

The present study aimed to explore the detection of IRLs, on variable MR scans, and describe the characteristics of IRLs presence in MS versus MS-mimics with highlighting the main findings. This study followed a systematic mapping review approach, to provide researchers and clinicians with a global picture of using IRs as an imaging biomarker in MS.

## 2. Materials and Methods

### 2.1. Search Strategy and Eligibility Criteria

This systematic mapping review was conducted on the detection of IRLs on MRI scans in MS for several reasons. The first is to overview the existing evidence on using IRLs as a proposed imaging biomarker in MS. The second is to describe the characteristics of IRLs found in MS versus MS-mimics. The third is to identify specific research topics in using IRLs as an MRI biomarker in MS for future systematic reviews and meta-analysis.

A systematic search was performed in January 2020 using the databases: PubMed, EMBASE and Web of Science to identify articles that evaluated the presence of IR in white matter lesions (WMLs) using MRI. The identified search terms were framed in PICO concepts. Patients/Population (P): multiple sclerosis OR MS. Intervention (I): magnetic resonance imaging OR MRI. Comparison (C) was not taken into account as there was not any comparison needed. Outcome (O): diagnosis OR differentiate. However, from this search, no results were generated. Thus, search using broader keywords was used in all the databases, including ((Multiple sclerosis OR MS) AND (rims OR rim) AND (lesion OR lesions)). The search strategy is provided in Supplementary Materials Figure S1. Review of the papers and analysis of results was conducted from January to July 2020.

### 2.2. Inclusion and Exclusion Criteria

The search was restricted to original articles on in vivo studies of human subjects published in peer-reviewed journals, written in English and featured an available full-text. The databases mentioned above were searched from 2001—the first date of using MRI in MS diagnosis. Grey literature was individually searched, including internet resources, theses, and conferences (Table 1). All duplications were removed using Mendeley.

### 2.3. Data Extraction

Relevant articles that fulfilled the inclusion and exclusion criteria (Table 1) were assessed by three reviewers (A.A., A.O., G.F.) independently. The complete extraction and assessment were conducted in three phases.

In the first phase, the three reviewers independently screened the titles and abstracts and removed the articles that did not fulfill the predefined inclusion/exclusion criteria.

In the second phase, all selected papers were reviewed by A.A. to select eligible studies for inclusion, as well as to perform a qualitative assessment. Papers were read and to determine if they contained data useful to the aim of this review. Disagreements between reviewers were resolved via discussion. The following details about each study were extracted by one reviewer (A.A.): study (author, date), type of the study, sample size, demographics, the aim of the paper, type of magnet strength, data analysis method, full MRI protocol procedure and main results and limitations.

In the third phase, a general quantitative overview of study characteristics was reported using descriptive statistics, median/mean and interquartile range (IQR) values. However, because of the heterogeneity of aims, the dataset used, techniques applied, and evaluation metrics specified in the selected publications, the results were stratified by pursued clinical aim/key findings.

## 3. Results

### 3.1. Literature Search and Study Characteristics

**Figure 1:**
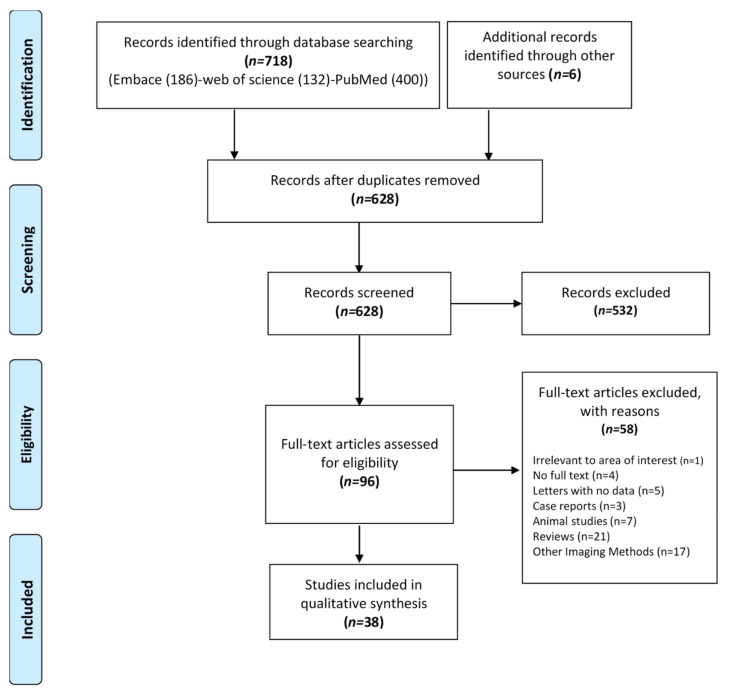
PRISMA flowchart of studies selection.

The literature search is illustrated in a flowchart (Figure 1). From the initial search and after removing duplicate studies, 628 articles were identified. Out of these, 527 articles were excluded as they did not meet the inclusion criteria. An additional 101 studies were excluded after reading the full text. Finally, a total of 38 studies were included in this systematic mapping review.

Of all the literature that was reviewed, seven studies (18%) were conference presentations/or posters. Six studies (15%) had a retrospective design, while the remaining 32 studies (84%) had a prospective design. Eleven studies were longitudinal with both retro/prospective design; 15 years’ follow up was the most prolonged study duration.

**Figure 2:**
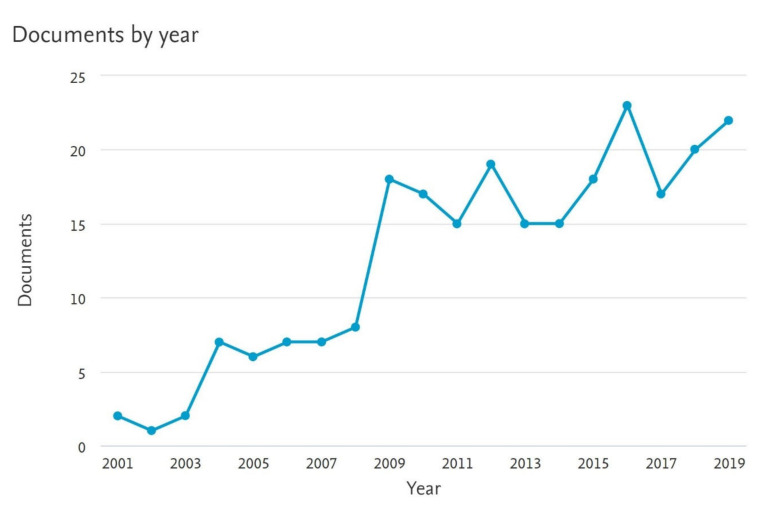
The graph illustrates the numbers of original articles on IRLs presence in MRI published annually, as indexed in Scopus (scopus.com); 2020 publications not included as not a full year.

All studies were published between February 2001 and January 2020. Figure 2 shows an increase in the number of published original studies on the topic of IRLs based on Scopus. 7T was used alone in 15 studies (39%), while 3T was used in 13 studies (34%) whereas four studies (11%) used both 3T and 7T. Two studies used 1.5T, one used 4.7T, while some studies did not provide this information.

Of the different MRI sequences used for white matter lesions, especially when detecting IRLs, Susceptibility weighted imaging (SWI) and T2*-Fluid-Attenuated Inversion Recovery (FLAIR) (combined) were used most often. In particular, SWI was combined with T2* and FLAIR in 52% of the studies. Additionally, SWI was combined with other sequences, such as T1-, T2-, and proton density-weighted images.

The aims and key findings of reported studies were subgrouped into (1) IRLs presence in MS subtypes, (2) the spatial distribution of white matter lesions with IR, (3) gender differences in the presence of IRLs, (4) the clinical relevance of IRLs (i.e., rim lesions linked with a disability and psychological impairment), (5) the prevalence of IRLs presence based on lesions and patients count (6), the evolution of IRLs over time, and (7) the pathological nature of IRLs. Due to the nature of this report, the results will be highlighted and discussed briefly.

#### 3.1.1. IRs in Patients with MS and MS Subtypes

Sixteen papers reported the presence of IRLs in MS subtypes [14,16,19,22,23,24,25,26,27,28,29,30,31,32,33,34]. Most of the publications examined patients with RRMS [22,24,29,31,32,35,36,37]. Three papers looked at RRMS and Secondary Progressive MS (SPMS) [29,31,35], while other studies compared MS with controls [14,16,22,27,31,32,35,36] or MS-mimics diseases [38,39]. The highest rate of IRL presence was found in RRMS (36%) followed by SPMS (27%) while the benign MS and clinically isolated syndrome (CIS) had the lowest percentage of (5%). One study each was found reporting on benign MS [32] and radiologically isolated syndrome (RIS) [34] while RRMS was most frequently reported in eight studies [22,23,24,26,29,31,32,36] followed by SPMS in six studies [16,23,28,29,30,31]. Interestingly, IRLs were absent in NMOSD [19], Susac’s syndrome [20] and ischemic lesions [21].

Llufriu et al. (2010) scanned 257 patients with the four MS subtypes; the IRLs detected were 7% in CIS, 11% in RRMS and 13% in SPMS with no rims detected in PPMS patients [30]. Similar work by Clarke et al. (2019) detected IRLs in 48% CIS, 59% RRMS and 39% SPMS [35]. In contrast, Chawla et al. (2016), scanning only nine patients (4 RRMS, 5 progressive) at 7T, and found that 5 out of 9 had IRLs, and 4 of these were progressive [19].

Although IRs are not commonly found in MS mimics [18,19,20], other brain disorders might have IR and IR have been reported to be present in brain abscess [38]. Clinically and radiologically they are not frequently encountered in the differential diagnosis of MS, but worth noting.

#### 3.1.2. IRLs and Lesion Localization

**Figure 3:**
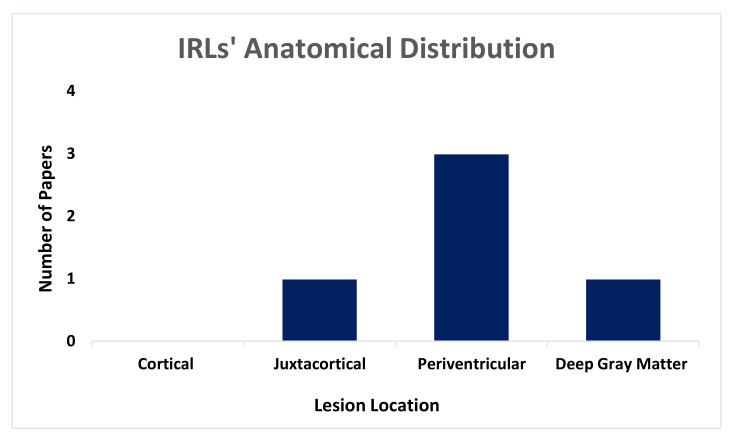
Illustrates the main anatomical location of the IRLs in MS lesions when reported (total of 5 papers).

Only four studies reported the anatomical location of the IRLs [22,32,35,40], indicating that periventricular was the common site and cortical area was the least for the rims to be present [22,32,35]. The IRLs were equally reported in both juxtacortical and DGM locations [40] (Figure 3). Dal-Bianco et al. (2019) showed that PRs were commonly seen in periventricular, while non-iron rims lesions were seen in DGM and juxtacortical locations [32].

#### 3.1.3. IRLs and Gender-specific Differences

Only seven papers reported the gender-specification related to IRLs presence. IRLs were reported to be more common in males and 70% more likely in young males [22,27,28,30,32,35,41]. Clarke et al. (2019) provided more details relating to gender-specific differences; suggesting male gender as the most important predictor of the proportion of IRLs in CIS and MS patients [35]. Males are 40% more likely to have IRs compared to females. Age seems to play a role with IR decreasing by 3% per year as age is increasing [35].

#### 3.1.4. Clinical Relevance of IRLs

**Figure 4:**
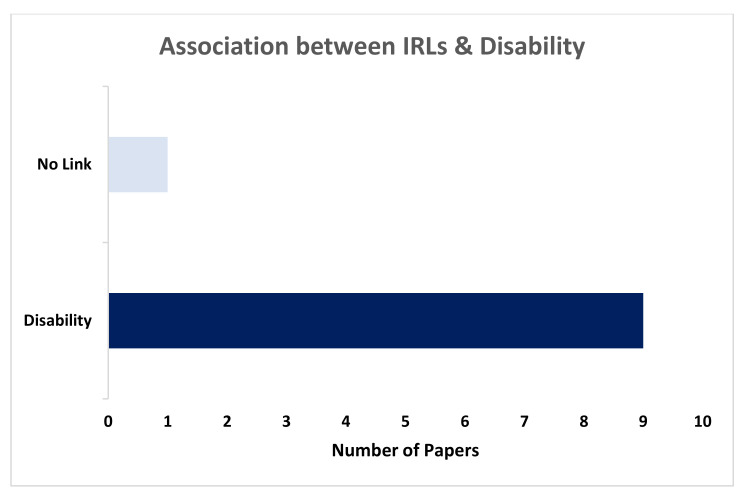
Illustrates the number of papers that found a link between IRLs presence and concurrent disability.

IRLs presence was related to poor cognitive performance and disability in 9 of 10 papers [14,17,27,29,32,34,40,41,42,43]. However, Kilsdonk et al. (2014) reported the lack of relationship between IRLs and physical disability [28]. This drawn conclusion was derived from scanning 33 patients with MS, 8 with rims, then were compared to 7 healthy controls at 7T (Figure 4). In contrast, Dal-Bianco et al. (2019) analysed 33 MS patients, 24 had IRLs, and the presence of IRLs was associated with higher FLAIR lesion load, which was related to poorer cognitive performance on the symbol digit modalities test [32]. No significant difference in disease progression was found between the two groups (IRLs and non-IRLs), four patients switched disease course during the study (3 RRMS into SPMS, 1 benign MS into RRMS), while three of these patients had IRLs.

#### 3.1.5. IRLs Prevalence

##### As a Proportion of Total White Matter Lesions’ Count

**Figure 5:**
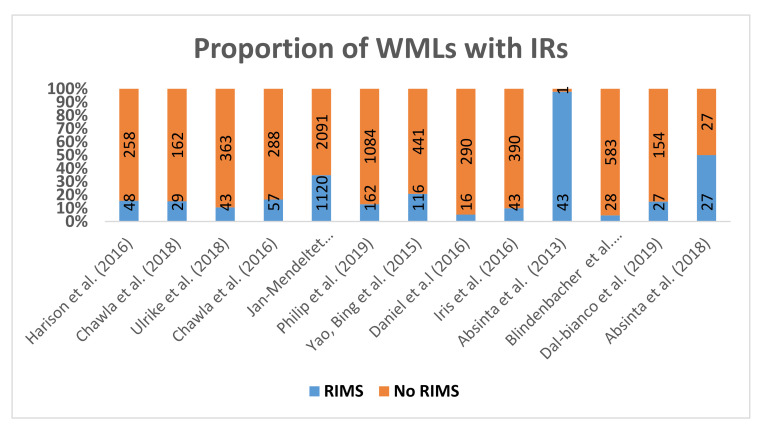
Illustrates the prevalence of Rim presence in MS patients, the percentage based on the total lesion number.

Figure 5 shows the proportion of lesions with IRs reported between studies. The total lesion counts across studies ranged from 44 to 3211 lesions. Four studies showed similar prevalence of IRLs presence, approximately ~10% [17,28,34,44]. However, Absinta et al. (2013) using 7T scanner found IRLs in acute gadolinium-enhancing lesions and found their presence in 75% (33/44 lesions), but in 2018 the same group reported in their 3T and 7T longitudinal study the presence of IR in chronic lesions was 50% (27/54 lesions) [45,46]. Both Danial and Blindenbacher shared a similar prevalence of 5% (16/306; 28/611 respectively) in acute lesions [17,34]. The MRI techniques used to detect rims in these studies were different, namely, Quantitative Susceptibility Mapping, (QSM), SWI, R2*, FLAIR with the mixed-use of both 3T and 7T MRI scanners.

##### As a Proportion of Patients Studied

**Figure 6:**
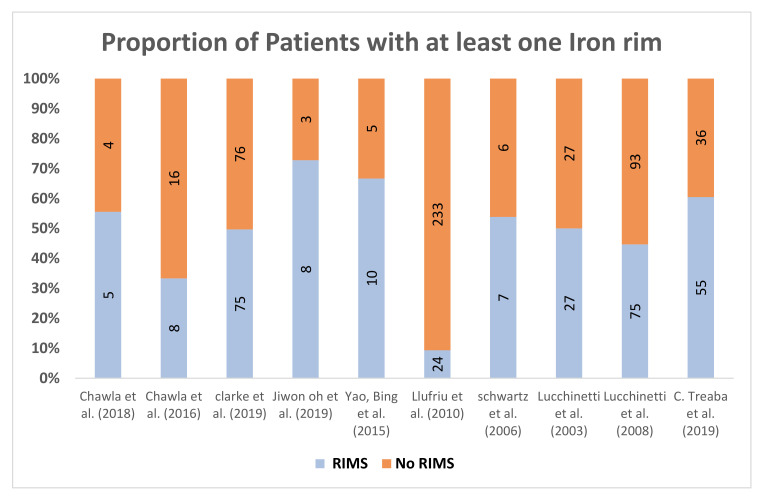
Illustrates the prevalence of IR detected in MS patients. Numbers of patients with at least one IR are shown.

Ten papers reported the proportion of patients with at least one IRL, the mean of the total number of MS patients was 97.3 (range 9–257) [29,31,35,38,47,48,49]. Eighty percent of the studies stated that rims could be detected in 50% to 70% of MS patients examined. Jiwon Oh et al. (2019) reported the highest percentage of IRLs presence which was 72% (8/11 MS patients) [50] while only 10%with IRs reported by Llufriu et al. (2010) scanning (24/233 MS patients) [30]. Similarly, Chawla et al. scanned nine patients (4 RRMS, 5 Progressive) at 7T, and 55.5% of patients had rims [19] (Figure 6). To sum up, histopathology and MRI study showed that 8–70% of MS lesions are surrounded by IRLs, which could be seen in both 3T and 7T [14,18].

#### 3.1.6. Rim Evolution Overtime

**Figure 7:**
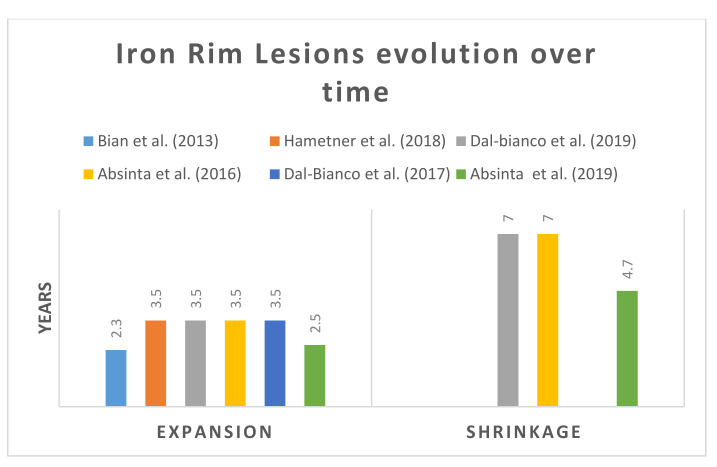
Shows in years the reported expansion of IRLs and shrinkage in MS.

Eleven papers reported whether the IRLs expand or shrink without accurately specifying the time frame [14,32,34,37,42,47,51,52,53,54,55]. However, six papers reported a different duration for expansion and shrinkage [12,14,18,32,42,52] (Figure 7). Longitudinal studies with a mean follow up of 3.5 years, observed slow expansion of IRLs that persist over time [14,47,52], then gradually the hypointense rim disappearing, returning to contrast NAWM after seven years [12,37]. IRLs are frequently detected in young lesions and early disease [24,56].

Some studies showed that rimmed lesions were larger and expanded by almost 29.33%. In comparison, lesions without rims were smaller in size by 10% [14]. IRLs significantly enlarge over time [14]. However, for non-rims, it has been reported that lesions disappear in a period of 2.5 to 4.7 years. In short, lesions with IRLs tend to grow slowly and without rim shrink over time.

An ECTRIMS presentation by Dal-Bianco et al. (2019) provided more insight into rim evolution [32]. They found that IRLs appear newly, enlarge slowly, then stabilize and gradually lose the iron rim. IRLs had a substantial larger initial volume and the rim became thinner after 3.5 years before partially disappear. The IRLs volume showed a gradual increase in size and fused with neighboring rim lesions within 3.5 years.

#### 3.1.7. Nature of Rims—Pathology

Rims likely reflect iron accumulation within a subset of macrophages/activated microglia at the lesion edge [42,52]. Lesions with IRLs are believed to be chronic active lesions, also known as smoldering or slowly expanding lesions (SELs), which fail to remyelinate and have subsequently been proposed to be a marker of tissue destruction, continuing inflammation and failure to repair [11,57].

Chronic active lesions are identified by gliotic, hypocellular centers and rims of activated microglia cells, macrophages and iron. However, these rims cannot be found in remyelinated or shadow plaques [58].

Pathological studies investigating the chronic MS lesions reported that up to 57% of these lesions are active or mixed [22,27,59]. The characterization of these lesions is attributed to the slow rate of increase in size and continuous tissue loss, which are commonly noticed in patients with long disease duration and progressive MS [14,22,27,59]. Chronic active lesions are commonly detected in patients with disease duration of 10 years or more and peak at 20 years [22].

Colm et al. [60] found that when comparing RRMS with PPMS patients, RRMS had higher numbers of SELs.

## 4. Discussion

This is the first systematic mapping review carried out on the presence of IRs in MS patients when using MRI. This review aimed to explore the detection of IRLs, on variable, clinical-quality MR scans, and describe the characteristics of IRLs presence in MS versus MS-mimics with highlighting the main findings. We primarily focused on study aims for which IRLs have been reported. Three different databases were searched, and after the screening, 38 studies were included in this review. Characteristics of the studies and IRLs were described.

In the past decade, several studies have focused on IRLs in MS and non-MS. Researchers around the world are working to exploit MRI data to improve MS diagnosis and treatment. Thus, an increase in the number of published IRLs studies was observed, reflecting the scientific community’s growing interest in the clinical value of IRLs presence in MRI.

Although T2*/FLAIR and phase imaging, including SWI, hold promise for identifying IRLs, yet there is no ideal technique to use in vivo. SWI is sensitive to iron detection which accumulates in normal-appearing brain tissue, in lesions, and the veins’ walls in MS patients [61,62,63]. IR is best appreciated on phase (rather than T2*) images [12]. However, for large lesion and vessel detection, Sati et al. suggested using T2* and FLAIR combined [64]. Many sequences can detect iron but one of the challenges when comparing IRLs studies in MS is the different MRI sequences used. Only by utilizing different sequences to the same individual can the value of each sequence be demonstrated. This has rarely been done in the reported studies. Based on the included studies, 7T was found to be a useful tool for tracking the evolution of MS lesions, especially concerning changes in iron content but 3T would be more practical to use in clinical settings [47]. This explains the higher number of 7T studies compared to 1.5T when exploring IRs.

The key findings from this review are that IRLs, which appear as dark, ring-like features on edges of some MS lesions, are believed to be chronic active lesions/slowly expanding lesions (SELs). These lesions fail to remyelinate and have subsequently been proposed to be a marker of tissue destruction, continuing inflammation and failure to repair [11,12]. Indeed, evidence from pathology and 7T MRI has shown that rimmed MS lesions were found to be significantly larger and underwent expansion by almost 30%, unlike non-rimmed lesions which reduced their size by 10% [14]. IRs were more frequently found in periventricular regions of young males with RRMS and linked with higher disability (EDSS score ≥5) [14,16] but appear absent in NMOSD [19], Susac´s syndrome [20] and ischemic lesions [21]. Thus, it seems that IRs have the potential to be a successful imaging marker for MS. More details about these results will be discussed in the next section.

### 4.1. The Presence and Spatial Distribution of IRLs

IRLs seem to be more common in RRMS; this might be due to the fact that RRMS patients were used more in studies compared to other MS subtypes. Besides, not all the studies included all MS subtypes in their studies. In contrast, two studies tested on four types of MS and both found IRLs were more common in SPMS than RRMS; however, the sample size was too small (9 patients) [65]. IRLs elevated in R2* were seen in both RRMS patients with low disability and those with long-standing SPMS [29]. A study found that 61% of RIS patients had at least one rim [40] 

It is hard to conclude from these studies as the heterogeneity of the sample size/MS types, as well as MRI scanners between studies, was high. However, the study by Schwart et al. compared IRLs presence in different diseases (glioma, metastases, abscess, MS) and found IRLs were more in abscess than MS; this could be from the higher number of abscess patients compared to MS (18,11 patients respectively) [38]. Interestingly, in terms of neuroinflammatory conditions, it seems IRLs are MS-specific as they are absent in NMO [19], Susac´s syndrome [20] and ischemic lesions [21].

Lastly, the periventricular region was the highest anatomical location of IRLs presence while the cortical was the least. This is to be expected as MS lesions are commonly seen around the ventricles [66]. Although the focus of this review was mainly in the brain, IRLs were also found in the spinal cord [40].

### 4.2. Gender Differences in IRLs

Few studies tested gender-specification of IRLs. Although IRLs could be detected in women, men had a higher prevalence, especially young men. Men are 10% to 40% more likely than women to have IRLs [35,41]. The reason is still unknown and needs further investigation.

### 4.3. Prevalence of IRLs

Different prevalence was reported of IRLs; the prevalence was calculated from the included studies based on lesions and patients counts. Overall, IRLs prevalence based on lesions counts ranges from 5–97% while 50–72% was based on patients’ numbers. The differences seen between the results could be explained by the different scanners/sequences used, which have a significant impact on lesion/rim detection. However, there is no clear conclusion about the exact IR prevalence in MS. Future studies calculating IRLs prevalence based on the number of patients would give a reasonable range compared to lesions count.

MRI ring-enhancing lesions are among the recognized patterns in MS and occur in one-quarter of all MRI enhancing lesions [30]. In contrast, few studies observed the peripheral hypointense rims on T2-weighted images. The relation of the ring enhancement with hypointense T2-weighted rims was found in 54% (7/13) of the included MS patients [19]. A pathological study investigating the correlation of biopsy MS subtypes with MRI reported that the hypointense T2-weighted rims were observed in 50% (27/54) of the cases [49].

### 4.4. The Evolution of IRLs over Time

The lesion volume of IRs showed a gradual increase in size and fusion with neighboring IR lesions within 3.5 years, and this was proven on both 3T and 7T studies. IRLs gradually decline over time and disappear in a few years.

IRLs expand in three to four years compared to non-IRLs, and the size stabilizes afterwards. IRLs had larger initial volumes and became thinner after 3.5 years and partially disappeared over time. New IRLs appear with an increased iron load of the entire lesion, transforming into IRLs within a year.

7T-MRI showed that the area around IRLs has a hyperintense signal, diffused and extended for several years. Although the nature of these changes is still unknown, 7T postmortem studies suggested that the hyperintensity signals could be an indication of demyelination and axonal degeneration. A study by Dal-Bianco et al. detected 9 of 16 hypointense lesions which formed as rims within 3.5 years. However, the other seven lesions started straight away, forming iron rims [67]. The explanation could be, there are two different types of rim developments, or they might miss the phase during the follow-up. To sum up, IRLs are a dynamic feature in MS, within seven years IRLs enlarge slowly, then stabilize and gradually disappear.

### 4.5. Pathological Nature of IRLs and Disability

Rims have a characteristic “pencil-thin” appearance at the junction of the lesion and adjacent normal-appearing WM. IR lesions are composed of iron-containing macrophages and microglia at the lesion edge. They display a phenotype of proinflammatory activation and in part contain early myelin degeneration products. IRLs have been suggested to label a subset of chronic active lesions [14,53,63]. Chronic lesion activity driven by smoldering inflammation is a pathological hallmark of progressive forms of MS. Pathological studies showed that the smoldering demyelination occurs in a similar extent in both PPMS and SPMS.

Assessing new lesions with 7T MRI indicates that a persistent phase rims had lower quantitative T1 signal intensities over time and could predict poor tissue outcomes. These interpretations were accordant with the pathological correlation of progressive MS and the concept of slowly expanding demyelination.

Recently, it has been recognized that iron and myelin are primarily contributing to WM MRI susceptibility. Iron has the properties of paramagnetic materials which allows a positive susceptibility for water content. However, myelin has the properties of diamagnetic materials which show negative susceptibility [58]. As a part of the demyelination process, it is believed that the inactive iron and proinflammatory microalga are obtained from the myelin and the debris of oligodendrocytes. Alternatively, another hypothesis states that the oligodendrocytes may contribute to releasing the iron in WM adjacent to plaques, which is thought to be produced by the inflammatory cytokines and discharged by microglia.

Hypointense rim lesions are promising predictors of the continuation of tissue injuries and inflammation in progressive MS. Thus, detecting the inflammation activity could be used to identify patients’ response to anti-inflammatory treatments. This might be valuable in improving disease prognosis as these lesions correlate with MS severity [68].

Disability scores tended to be worse in patients with rims. Despite that, the exact clinical significance of rim lesions in MS is still not clear.

This study has some interpretations of risks and limitations. One of the risks affecting all systematic mapping reviews is related to selective reporting bias [69]. To minimize this risk, three different databases were used which provide a comprehensive list of articles covering the various aspects of this mapping review. It is also worth noting that although it was decided to exclude reviews from the study however, the reference list for the reviews was checked and all relevant papers were included. Another selection bias risk relates to the criteria used to select the articles to be analyzed during the study. To mitigate such a risk, both the inclusion and exclusion criteria were clearly defined. One of the limitations is the limited number of papers available covering the same topic of interest of this review. There was much heterogeneity between studies, for example, different MRI magnetic strength, different aims, different sample size and disease types. Additionally, not all studies reported the needed information.

## 5. Conclusions

Based on the results of the present study it seems that IRLs were more frequently found in periventricular regions of young males with RRMS. Additionally, IRLs could be linked with a higher disability and appear to be absent in MS-mimicking disorders. Different prevalence was reported of IRLs; the prevalence was calculated from the included studies based on lesions and patients counts. (5–97%, 50–72% respectively). The lesion volume of IRs showed a gradual increase in size and fusion with neighboring IR lesions within 3.5 years. Pathologically, IRLs are a sign of chronic active inflammation and persisting demyelinating activity. Although IRs have a promising potential to be a proposed diagnostic imaging biomarker and disease progression, there is still much to learn about the aetiology and mechanisms underlying IRLs, especially regarding the link between IRLs and clinical impact and IRLs evolution and prevalence. To answer these questions, more extended observation in larger cohorts is required. Meta-analysis may further be considered.

## Figures and Tables

**Table 1:** This table illustrates the study selection criteria.

Inclusion	Exclusion
Brain studies on WMLs with rims presence	Studies published before 2001
Pathological studies	Animal studies
Studies published in peer-review journals or the grey literature	Case reports, reviews or systematic literature reviews and qualitative studies, opinion pieces, editorials comments, and news
Literature is written in English MRIs field strength reported	

## Supplementary Materials

For the study protocol check: https://rdmc.nottingham.ac.uk/handle/internal/8308.
